# Purification and characterization of crude fructooligosaccharides extracted from red onion (*Allium cepa* var. *viviparum*) by yeast treatment

**DOI:** 10.1186/s12934-023-02289-7

**Published:** 2024-01-10

**Authors:** Jakkrit Aisara, Jirat Wongsanittayarak, Nalapat Leangnim, Kraikrit Utama, Padchanee Sangthong, Woraprapa Sriyotai, Sugunya Mahatheeranont, Suphat Phongthai, Kridsada Unban, Saisamorn Lumyong, Chartchai Khanongnuch, Pairote Wongputtisin, Apinun Kanpiengjai

**Affiliations:** 1 Program in Biotechnology, Multidisciplinary and Interdisciplinary School, Chiang Mai University, Chiang Mai, 50200 Thailand; 2 Division of Biochemistry and Biochemical Innovation, Department of Chemistry, Faculty of Science, Chiang Mai University, Chiang Mai, 50200 Thailand; 3 Office of Research Administration, Chiang Mai University, Chiang Mai, 50200 Thailand; 4 Department of Chemistry, Faculty of Science, Chiang Mai University, Chiang Mai, 50200 Thailand; 5 Division of Food Science and Technology, School of Agro-Industry, Faculty of Agro-Industry, Chiang Mai University, Chiang Mai, 50200 Thailand; 6 Division of Microbiology, Department of Biology, Faculty of Science, Chiang Mai University, Chiang Mai, 50200 Thailand; 7 Department of Biology, Faculty of Science, Chiang Mai University, Chiang Mai, 50200 Thailand; 8 Program in Biotechnology, Faculty of Science, Maejo University, Chiang Mai, 50200 Thailand; 9 Academy of Science, The Royal Society of Thailand, Bangkok, 10300 Thailand

**Keywords:** Purification, Fructooligosaccharides, Yeast treatment, *Allium cepa* var. *viviparum*

## Abstract

**Background:**

Yeast treatment has been used for purification of fructooligosaccharides (FOSs). However, the main drawback of this approach is that yeast can only partially remove sucrose from crude FOSs. The main objective of this research was to screen yeast strains for the capability of selectively consuming unwanted sugars, namely fructose, glucose, and sucrose, in crude FOSs extracted from red onion (*Allium cepa* var. *viviparum*) with minimal effect on FOS content.

**Results:**

Among 43 yeast species isolated from Miang, ethnic fermented tea leaves, and Assam tea flowers, *Candida orthopsilosis* FLA44.2 and *Priceomyces melissophilus* FLA44.8 exhibited the greatest potential to specifically consume these unwanted sugars. In a shake flask, direct cultivation of *C*. *orthopsilosis* FLA44.2 was achieved in the original crude FOSs containing an initial FOSs concentration of 88.3 ± 1.2 g/L and 52.9 ± 1.2 g/L of the total contents of fructose, glucose, and sucrose. This was successful with 93.7% purity and 97.8% recovery after 24 h of cultivation. On the other hand, *P*. *melissophilus* FLA48 was limited by initial carbohydrate concentration of crude FOSs in terms of growth and sugar utilization. However, it could directly purify two-fold diluted crude FOSs to 95.2% purity with 92.2% recovery after 72 h of cultivation. Purification of crude FOSs in 1-L fermenter gave similar results to the samples purified in a shake flask. Extracellular β-fructosidase was assumed to play a key role in the effective removal of sucrose. Both *Candida orthopsilosis* FLA44.2 and *P*. *melissophilus* FLA44.8 showed γ-hemolytic activity, while their culture broth had no cytotoxic effect on viability of small intestinal epithelial cells, preliminarily indicating their safety for food processing. The culture broth obtained from yeast treatment was passed through an activated charcoal column for decolorization and deodorization. After being freeze dried, the final purified FOSs appeared as a white granular powder similar to refined sugar and was odorless since the main sulfur-containing volatile compounds, including dimethyl disulfide and dipropyl trisulfide, were almost completely removed.

**Conclusion:**

The present purification process is considered simple and straight forward, and provides new and beneficial insight into utilization of alternative yeast species for purification of FOSs.

**Supplementary Information:**

The online version contains supplementary material available at 10.1186/s12934-023-02289-7.

## Background

Prebiotics have been defined as selectively fermented ingredients that allow for specific changes, both in the composition and/or activity in the gastrointestinal microflora, that can confer benefits upon the well-being and health of the host. It is well known that most oligosaccharides are known to possess prebiotic properties. Fructooligosaccharides (FOSs) are the first accepted oligosaccharide prebiotics according to their properties that meet the criteria for prebiotic classification, such as resistance to gastrointestinal conditions and absorption, as well as their selective stimulation of bifidobacteria and lactobacilli in the large intestine [1]. In addition, FOSs provide other functional properties that can promote human health such as by enhancing absorption of essential minerals and ions in the small intestine, regulating glucose metabolism in the blood, lowering levels of triglycerides, phospholipids, and cholesterol, as well as by further reducing the risk of colon cancer [2]. Although FOSs are well recognized, their production process has been extensively studied and developed for the past several decades.

FOSs can be produced by direct extraction from plant materials, synthesis from sucrose by enzymatic and/or microbial transformation methods, and by enzymatic degradation of inulin and fructan [3]. The simplest production process involves the direct extraction of FOSs from plants such as onions, artichokes, chicory, and asparagus [4]. Our previous work has revealed that red onions (*Allium* cepa var. *viviparum*) are a potential source of inulin-neo-FOSs. The neokestose obtained from red onions can efficiently and specifically stimulate the population of *Bifidobacterium breve* in infant gut microbiota [5]. Notably, a previously published study has reported that neo-FOSs possess a great degree of thermostability and chemical resistance [6]. Nevertheless, FOSs obtained by this process and other previously mentioned processes generally consist of unwanted sugars, namely fructose, glucose, and sucrose, which consequently require removal steps to achieve purified FOSs. The occurrence of unwanted sugars in FOSs reduces the prebiotic effect of FOSs and increases plasma glucose levels, as they are ingestible and absorbable in the gastrointestinal system. Unpurified FOSs products can be a risk factor of metabolic disorders, such as obesity and diabetes [7], and can limit their application in overweight and obese people.

Several physicochemical strategies have been established for FOS purification including chromatography [8] and membrane reactor [9]. These strategies are high-cost production processes that are operated under complicated conditions. Microbial treatment is an alternative and promising strategy since the unwanted sugars can be selectively removed from crude FOSs with a minimum effect on degradation of FOSs. In addition, it can be a simple purification process when specific and non-fastidious microorganisms are used. These microorganisms can specifically assimilate and/or ferment the unwanted sugars present in crude FOSs without any additional growth factor supplementation. Notably, these aforementioned strategies have not yet been employed for purification of FOSs derived from natural sources. The most current report on the purification of FOSs from a natural source has been achieved using activated charcoal for the purification of FOSs extracted from garlic acid [10]. With regard to the potential limitations of this physicochemical strategy for the purification of FOSs, microbial treatment of red onion extract would be of further interest. To date, most studies focus on yeast for FOS purification, specifically *Pichia pastoris* GS115 [11], *Wickerhamomyces anomala* XS1 [12], and *W. anomala* GXL-22 [13]. Recently, there has been an attempt to identify a potentially beneficial application of *Bacillus coagulan* DSM1 and *Bc.* (*Bacillus*) *subtilis* YBJ in the purification of FOSs [14]. The major drawback of these organisms is their lack of efficiency in sucrose assimilation and fermentation. Sucrose, as opposed to glucose and fructose, is always retained after cultivation of crude FOSs by the previously mentioned yeast strains and bacteria, resulting in the degree of purity of FOSs ranging from 55 to 85%. To improve the purity, it is essential to add other steps of purification. For example, the addition of β-fructosidase after cultivation of *Bc. coagulans* could transform the sucrose residues into FOSs. With regard to this process, FOSs yield was increased with an elevated FOSs purity of up to 92.1% [15]. Generally, *Saccharomyces cerevisiae* produces potential β-fructosidase that can hydrolyze sucrose and FOSs with regard to its *SUC2* gene. Co-fermentation of an FOS producer, *Aspergillus ibericus*, and the genetic engineered *S. cerevisiae* YIL62W with *SUC2* gene disrupted could improve the purity of FOSs to 97.4% [16]. Screening of microorganisms with high efficacy in the removal of the unwanted sugars is relevant to the current drawback of FOSs purification and may provide a less complicated purification process than that which has already been reported. The main objective of this research study was to screen yeast strains isolated from Miang, traditionally fermented Assam tea leaves (*Camellia sinensis* var. *assamica*) of northern Thailand [17], and flowers of Assam tea [18], for selective removal of the unwanted sugars present in crude FOSs obtained from red onions. The reaction mechanisms associated with the purification of FOSs by the selected yeast strains have also been proposed. Furthermore, safety assessments of the selected yeast strains, as well as any additional purification and formulation steps needed to achieve purified FOSs, have also been described. Overall, this research study has presented a complete process scheme for the purification and formulation of FOSs.

## Results

### Properties of red onion extract

**Fig. 1:**
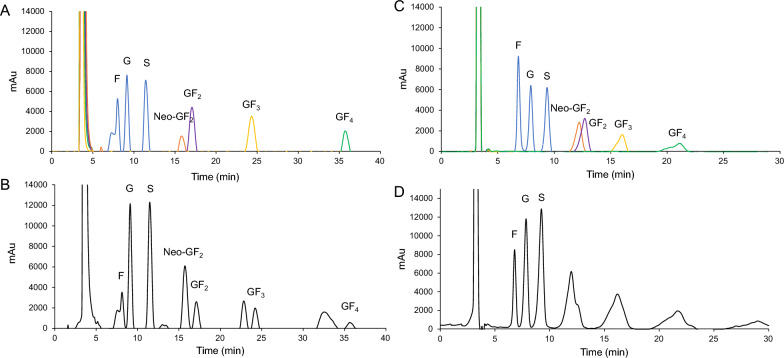
HPLC chromatograms of **A** standards at 1000 ppm and **B** crude FOSs when analyzed by a Shodex HILICpak VN-50 4D column with a mobile phase consisting of acetonitrile and deionized water at a ratio of 80: 20 (v/v). HPLC chromatogram of **C** standards at 1000 ppm and **D** crude FOSs when analyzed by a Shodex Asahipak NH_2_P-50 4E column with a mobile phase consisting of acetonitrile and deionized water at a raio of 75: 25 (v/v). Fructose, F; glucose, G; sucrose, S; Neokestose, neo-GF_2_; kestose, GF_2_; nystose, GF_3_; fructofuranosyl nystose; GF_4_

The red onion extract appeared clear with a pinkish purple color. After removing the outer layer of 10 kg red onion, a total of 8.8 kgs of peeled red onions were obtained. They were then chopped, homogenized, and extracted to achieve 1200 mL of red onion extract, which consisted of 16.18% w/v of the total soluble solid content. Carbohydrates (90.54% w/w) and proteins (7.73% w/w) were found to be the major and minor constituents, respectively while the others included crude fiber (0.88% w/w), crude fat (0.61% w/w), and minerals (0.01% w/w). The HPLC chromatogram demonstrates the clearly distinguished peaks of glucose, fructose, sucrose, kestose (GF_2_), neokestose (neo-GF_2_), nystose (GF_3_), and fructofuranosyl nystose (GF_4_) (Fig. 1). The red onion extract contained 135.79 g/L total carbohydrates, which was composed of fructose (7.67 g/L), glucose (21.34 g/L), sucrose (14.32 g/L), and other FOSs that included kestose (GF_2_, 4.47 g/L), neokestose (neo-GF_2_, 14.07 g/L), nystose (GF_3_, 10.44 g/L), fructofuranosyl nystose (GF_4_, 12.71 g/L), and other saccharides (50.77 g/L). Here, it could be concluded that the red onion extract contained 41.7 g/L of total detectable FOSs and 50.8 g/L of the other unidentified carbohydrates. Neokestose was the major FOSs constituent found in the red onion extract. Based on 100 g of fresh red onions, each carbohydrate has been presented in Table 1. Since FOSs are the main carbohydrates in the red onion extract, they were assigned to crude FOSs in this study.

**Table 1:** Carbohydrate and FOSs contents of red onion extracts

Carbohydrates	g/L	g/100 g fresh weight ^b^
Total carbohydrates	135.79 ± 4.95	18.52 ± 0.67
Fructose	7.67 ± 0.48	1.05 ± 0.07
Glucose	21.34 ± 1.04	2.91 ± 0.14
Sucrose	14.32 ± 1.04	1.95 ± 0.14
Neokestose (Neo-GF_2_)	14.07 ± 1.08	1.92 ± 0.15
Kestose (GF_2_)	4.47 ± 0.24	0.61 ± 0.03
Nystose (GF_3_)	10.44 ± 0.77	1.42 ± 0.11
Fructofuranosyl nystose (GF_4_)	12.71 ± 1.68	1.73 ± 0.23
Other saccharides^a^	50.77 ± 1.4	6.92 ± 0.19

^a^ Other saccharide contents were calculated from the differences between total carbohydrates and total amounts of fructose, glucose, sucrose, neokestose, kestose, nystose, and fructofuranosyl nystose

^b^ g/100 g fresh weight was calculated as a percentage of total mass of each carbohydrate in relation to total mass of peeled fresh red onions

### Screening of yeast strains for selective removal of fructose, glucose, and sucrose

**Fig. 2:**
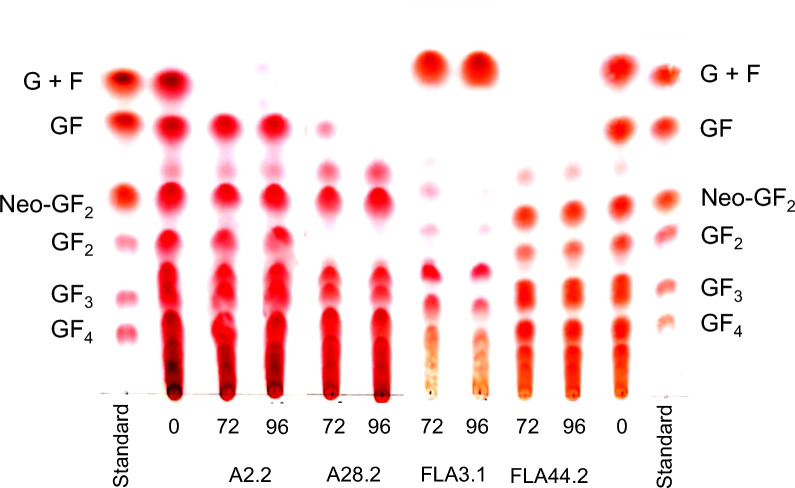
Examples of different FOSs profiles obtained from cultivation of a representative yeast strain in two-fold diluted crude FOSs extracted from red onions at 30 °C for 72 and 96 h. Profiles have been categorized into four groups based on their ability for sugar consumption. **A** Group 1: *Pichia manshurica* A2.2 could apparently consume only glucose and fructose. **B** Group 2: *Debaryomyces hansenii* A28.2 could consume glucose, fructose, sucrose, and neokestose. **C** Group 3: *Pseudozyma hubeiensis* FLA3.1 could utilize fructose, glucose, sucrose, and FOSs. **D** Group 4: *Candida orthopsilosis* FLA44.2 could selectively consume fructose, glucose, and sucrose

A total of 43 yeast species isolated from Miang and Assam tea flowers were screened for the possibility of being used in the selective removal of fructose, glucose, and sucrose with minimal effect on the FOSs present in red onion extracts (Additional file 1: Table S1). Various FOSs profiles of sugar utilization were generated after the cultivation of each yeast species in the crude FOSs (Additional file 1: Fig. S1) and could be categorized into four groups (Fig. 2) as follows: group 1 consisted of 19 yeast species that could apparently utilize only glucose and fructose, thus sucrose and FOSs remained after cultivation; group 2 consisted of 7 yeast species that were similar to group 1, but they were able to further consume kestose; group 3 consisted of 15 yeast species that could utilize fructose, glucose, sucrose, and FOSs; group 4 consisted of two yeasts species isolated from Assam tea flowers, namely *Candida orthopsilosis* FLA44.2 and *Priceomyces melissophilus* FLA48. These yeast strains selectively removed fructose, glucose, and sucrose and were presumptively identified as yeast species with the potential to purify FOSs but would require confirmation in further experiments.

### Characteristics of selected yeast strains during cultivation in red onion extract

**Fig. 3:**
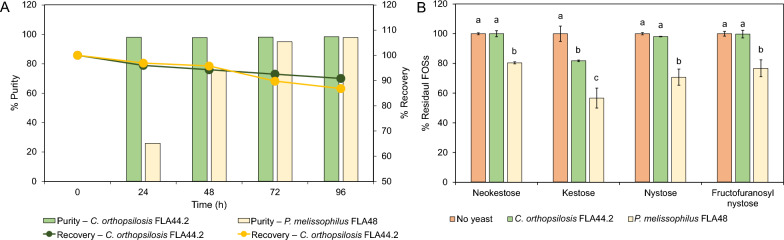
Primary screening results for the purification of the two-fold diluted crude FOSs by *Candida orthopsilosis* FLA44.2 and *Priceomyce melisophilus* FLA48. **A** Percentages of purity and recovery of FOSs. **B** Residual FOSs after 96 h of yeast cultivation ranging from neokestose, kestose, nystose, and fructofuranosyl nystose

During the confirmation step, it was revealed that *C*. (*Candida*) *orthopsilosis* FLA44.2 exhibited higher efficacy in terms of the removal of the unwanted sugars present in the two-fold diluted FOSs than *P*. (*Priceomyces*) *melissophilus* FLA48 for the purity and recovery of FOSs. *Candida orthopsilosis* was able to purify crude FOSs at a purity value of 97.9% and a recovery value of 96.0% after 24 h of cultivation. Longer periods of cultivation led to a lower degree of percentage recovery; however, the degree of purity was slightly higher (Fig. 3A). During the course of purification, *P*. *melissophilus* retained 89.7% of purified FOSs with a purity value of 94.9% after 72 h of cultivation. The retained amounts of FOSs, specifically kestose, neokestose, nystose, and fructofuranosyl nystose, after 96 h of cultivation for each yeast strain, are shown in Fig. 3B. It was found that 20% of the initial kestose content was decreased after cultivating the crude FOSs by *C*. *orthopsilosis* FLA44.2, while no significant differences of the initial neokestose, nystose, and fructofuranosyl nystose contents were observed. All analyzed initial FOSs contents were reduced after cultivation of *P. melissophilus* FLA48.

Based on the FOSs profile, β-fructosidase is the key enzyme that plays an important role in FOSs hydrolysis during the purification of crude FOSs. *Saccharomyces cerevisiae* is naturally a β-fructosidase producer, thus being able to hydrolyze FOSs and can be considered a positive control. The activity of β-fructosidase was found in both the cell and extracellular fractions of the crude FOSs cultured by *C*. *orthopsilosis* FLA44.2 and *P*. *melisophilus* FLA48. However, the quantity was obviously lower than that which was detected from the cells and culture supernatant of *S*. (*Saccharomyces*) *cerevisiae* (Table 2). The highest degree of extracellular β-fructosidase produced by *C*. *orthopsilosis* FLA44.2 was found at 24 h of cultivation and was then decreased and undetectable after 48 h of cultivation, while the cell bound enzyme was detected throughout the cultivation process. Extracellular β-fructosidase activity was observed during the initial stage until the end of the cultivation process of *P. melissophilus* FLA48 in the crude FOSs. The highest degrees of activity were observed during 48 and 72 h of cultivation, which were the periods of time that indicated the highest degree of purity of FOSs. Furthermore, *P*. *melissophilus* FLA48 could extensively produce cell bound β-fructosidase.

**Table 2:** Extracellular and cell-bound β-fructosidase activities of *Saccharomyces cerevisiae*, *Candida orthopsilosis* FLA44.2, and *Priceomyces melissophilus* FLA48 during the fermentation of crude FOSs at 30ºC for 96 h

I) Extracellular enzyme (mU/mL)			
Time (h)	*S. cerevisiae*	*C. orthopsilosis* FLA44.2	*P. melissophilus* FLA48
24	148.8 ± 3.3	19.3 ± 5.6	9.5 ± 1.1
48	255.4 ± 3.4	8.3 ± 1.1	12.6 ± 3.9
72	245.1 ± 4.4	nd	10.2 ± 1.7
96	218.5 ± 4.3	nd	7.1 ± 0.0

II) Cell-bound enzyme (mU/mL)			
Time (h)	*S. cerevisiae*	*C. orthopsilosis* FLA44.2	*P. melissophilus* FLA48
24	215.4 ± 5.5	9.9 ± 3.4	2.0 ± 1.1
48	171.5 ± 3.4	11.4 ± 2.3	2.8 ± 1.1
72	152.6 ± 3.7	24.4 ± 3.3	16.6 ± 1.7
96	187.6 ± 4.7	17.8 ± 3.3	9.1 ± 3.3

*nd* not detected

### Safety of selected yeast strains

**Fig. 4:**
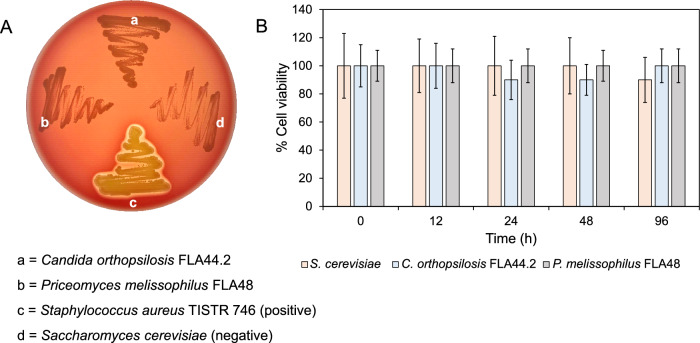
Safety evaluation of *Candida orthopsilosis* FLA44.2 and *Priceomyces melissophilus* FLA48. **A** Hemolysis production where *Staphylococcus aureus* TISTR 746 was used as the positive control and *Saccharomyces cerevisiae* was used as the negative control. **B** Cytotoxic effect of different cultures of *Candida orthopsilosis* FLA44.2 and *Priceomyces melissophilus* FLA48 on the viability of the small intestinal epithelial cell line Different lowercase letters indicate significant differences at *p* < 0.05 when compared to the control (no yeast).

Two selected yeast strains were assessed for their safety by employing two different methods. Hemolytic activity was investigated at 30 °C for 96 h, which represented the appropriate cultivation conditions for microbial FOS purification. *Staphylococcus aureus* TISTR 746 indicated positive results characterized as β-hemolysis with regard to 1–2 mm clear zone formation around the colony. No clear zone formation was found around the colonies of *C*. *orthopsilosis* FLA44.2 and *P*. *melissophilus* FLA48, which did agree with the negative control (Fig. 4A). Yet, hemolytic activity produced by these strains was negative and referred to as γ-hemolysis. In addition, the culture supernatant of the yeast strains grown in crude FOSs was assessed by the cytotoxicity test. Here, the small intestinal epithelial cell line was used as the representative cell line for the assessment. The results clearly indicate that there were no significant differences among the cells treated with the culture supernatant collected from 0 to 96 h of the cultivation of *C*. *orthopsilosis* FLA44.2 and *P*. *melissophilus* FLA48 in the crude FOSs (Fig. 4B). The results were in accordance with those attributed to the negative control (*S*. *cerevisiae*).

### Efficiency of selected yeast strains in the removal of fructose, glucose, and sucrose

**Fig. 5:**
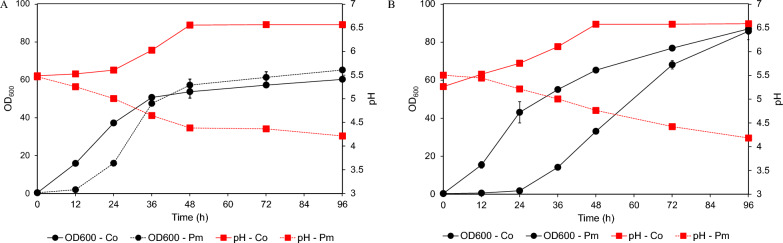
Profiles of OD_600_ and pH values of *Candida orthopsilosis* FLA44.2 (Co) and *Priceomyces melissophilus* FLA44.2 (Pm) recorded during the process of cultivation in 250 mL Erlenmeyer flasks containing (**A** two-fold diluted crude FOS and **B** crude FOSs at original concentrations

Regarding their ability to grow in the two-fold diluted crude FOSs of the selected yeasts, it is highly possible that they could also directly grow in the original concentration of the crude FOSs. The selected yeast strains were cultivated in a 250-mL shake flask containing an original concentration of the crude FOSs when compared with the two-fold diluted crude FOSs. The results indicate that both strains were able to directly grow in the crude FOSs at the original concentration. Remarkably, *C*. *orthopsilosis* FLA44.2 exhibited a more rapid degree of growth in the crude FOSs than *P*. *melissophilus* FLA48 due to the short lag phase (Fig. 5A and B). A specific growth rate of 0.23 h^−1^ was observed in the diluted crude FOSs and 0.22 h^−1^ in the original concentration of the crude FOSs, which were approximately two times higher than those of *P*.* melissophilus* FLA48 under the same culture conditions (Table 3). There was no effect of initial sugar concentrations on the growth of *C*. *orthopsilosis* FLA44.2. However, this effect occurred when *P*. *melissophilus* FLA44.2 was cultured in the original concentration of the crude FOSs. The specific growth rate of *P*. *melissophilus* FLA44.2 was decreased to 0.09 h^−1^ and its lag phase was extended from 12 to 24 h. Accordingly, the pH profile during cultivation of both yeasts was distinctively different. *Candida orthopsilosis* FLA44.2 altered the pH value of the crude FOSs culture from pH 5.5 to pH 6.5 as opposed to *P. melissophilus* FLA48, which tended to decrease the pH value of the crude FOSs culture from pH 5.5 to pH 4.3. *Candida orthopsilosis* FLA44.2 exhibited significant potential in terms of the ability to remove unwanted sugars. It could effectively remove fructose, glucose, and sucrose in the diluted crude FOSs within 24 h with a purity value of 96.6% and a FOSs recovery value of 96.4% (Table 3). When the cultivation was extended up to 96 h, the FOSs purity was reduced, as has been previously reported. With a similar degree of purity and recovery, *P*. *melissophilus* FLA48 required 96 h of cultivation to achieve the highest degree of purity for FOSs; however, the degree of FOSs recovery was slightly lower than that of *C*. *orthopsilosis* FLA44.2 (89.8%). At the original concentration of the crude FOSs, *C*. *orthopsilosis* FLA44.2 still exhibited the greatest ability to remove unwanted sugars at values as high as 93.7% purity of FOSs, along with a recovery value of 97.8%, after 24 h of cultivation. On the other hand, *P*. *melissophilus* FLA48 was able to partially purify the original concentration of the crude FOSs. After 96 h of cultivation in the crude FOSs, the highest purity of FOSs was obtained at 76.3%.

**Table 3:** Growth parameters of *Candida orthopsilosis* FLA44.2 and *Priceomyces melissophilus* FLA48 during fermentation of crude FOSs with and without dilution in both shake flask and fermenter scales

Scale	Yeast strains	Dilution	μ (h^−1^)	% Purity					% Recovery				
				24	36	48	72	96	24	36	48	72	96
Flask	*C. orthopsilosis* FLA44.2	Two-fold	0.23	96.6	97.7	98.4	98.4	98.5	96.4	96.7	93.6	92.9	93.0
		No	0.22	93.7	98.0	98.3	99.1	99.2	97.8	96.4	95.1	93.9	93.8
	*P. melissophilus* FLA48	Two-fold	0.13	27.3	58.8	77.9	95.2	96.9	93.2	94.4	93.0	92.2	89.8
		No	0.09	3.0	10.5	27.4	72.0	76.3	96.2	94.1	91.6	92.1	91.2
Fermenter	*C. orthopsilosis* FLA44.2	No	0.21	91.6	96.4	98.2	98.2	98.2	96.3	93.2	92.3	87.8	89.3
	*P. melissophilus* FLA48	Two-fold	0.12	9.0	65.1	79.0	96.7	97.1	88.7	87.3	87.7	85.6	85.0

Initial concentrations of the original FOSs and two-fold diluted FOSs were 88.3 ± 1.2 g/L and 47.9 ± 1.5 g/L, respectively

**Fig. 6:**
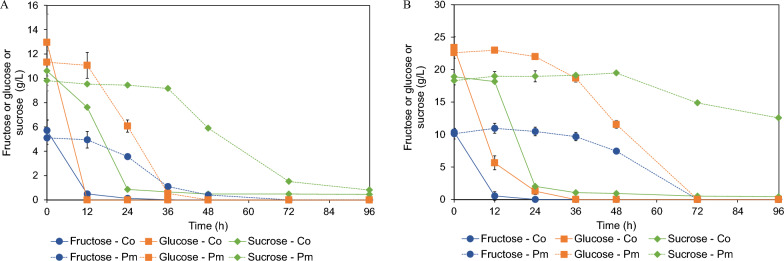
Profiles of fructose, glucose, and sucrose recorded during cultivation of *Candida orthopsilosis* FLA44.2 (Co) and *Priceomyces melissophilus* FLA44.2 (Pm) during the process of cultivation in 250 mL shake flasks containing **A** two-fold diluted crude FOS and **B** crude FOSs at original concentrations

**Fig. 7:**
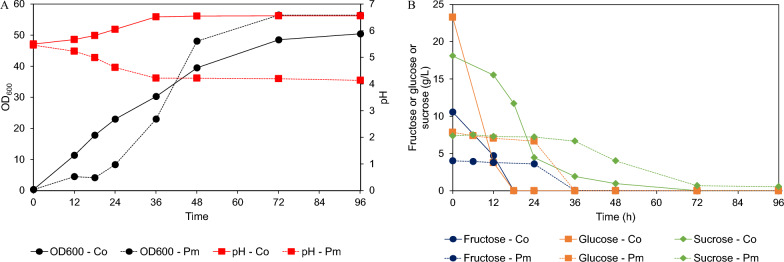
**A** Profiles of OD_600_, as well as pH values and **B** profiles of fructose, glucose, and sucrose, recorded during cultivation of *Candida orthopsilosis* FLA44.2 (Co) and *Priceomyces melissophilus* FLA44.2 (Pm) in a 1-L stirred tank fermenter containing original concentrations of the crude FOSs and two-fold diluted crude FOS, respectively

Considering the changes in the unwanted sugars, it was revealed that both yeast strains exhibited a similar sugar utilization pattern. Fructose and glucose, as simple sugars, were directly utilized by either assimilation or fermentation. After they had completely disappeared as a result of cultivation, sucrose contents tended to decrease (Fig. 6A and Fig. 6B). This phenomenon was observed to be in accordance with the profile of extracellular β-fructosidase, which was detected during the purification of FOSs, implying that this enzyme plays a key role in the removal of sucrose. According to these results, it could be concluded that *C*. *orthopsilosis* FLA44.2 could directly purify the original concentration of FOSs and it is necessary to dilute crude FOSs before purification using *P. melissophilus* FLA48. Purification of crude FOSs in 1-L fermenter produced similar results to that which was purified in the shake flask in terms of the purity, recovery, and profile of the unwanted sugar removal (Fig. 7A, B).

### Decolorization and deodorization of FOSs purified by activated charcoal

The purified FOSs obtained from the cultivation of each yeast strain was prepared and applied to an activated charcoal column to remove any unpleasant color and odor, and the results are shown in Table 4. The chromatographic method recovered 94.8% and 92.8% of the FOSs that were previously purified by *C. orthopsilosis* FLA44.2 and *P. melissophilus* FLA48, and these values were equivalent to 82.8% and 78% of the initial FOS contents, respectively. No fructose, glucose, and sucrose were detected after HPLC analysis, indicating that the FOSs was completely purified. After being freeze dried, the obtained FOS powders appeared as a white fine powder (Additional file 1: Fig. S2) that was slightly greener (-Δa* values) and yellower (-Δb* values) than the refined sugar, which served as the control. Their L*, a*, b* values were closely related to the refined sugar. Sulfur-containing volatile compounds, as the major ingredients causing the unpleasant odor in onions, were investigated and have been described in Additional file 1: Table S3. Volatile compounds, including dimethyl disulfide, methyl propyl disulfide, methyl 1-propenyl disulfide, and dipropyl trisulfide, were mainly found in the crude FOSs. After purification of the crude FOSs by selected yeast strains and chromatographic methods, only levels of dimethyl disulfide and dipropyl trisulfide were obviously reduced, while others remained stable. However, the purified FOSs were odorless when compared with the crude FOSs powder.

**Table 4:** Summary purification of FOSs obtained from red onion extract using yeast treatment and the chromatographic method

I.) *C. orthopsilosis* FLA44.2						
Sample	Total FOSs (g/L)	Purity	Recovery (%)	L*	a*	b*
Crude FOSs	88.3 ± 1.2	–	100.0	76.92 ± 0.18	10.78 ± 0.09	2.33 ± 0.05
Biological treatment	77.1 ± 0.3	98.2	87.3	77.46 ± 0.1	1.90 ± 0.06	9.88 ± 0.27
Chromatography	73.1 ± 5.4	100.0	82.8	95.61 ± 0.29	− 1.76 ± 0.03	3.76 ± 0.07

II.) *P. melissophilus* FLA48						
Sample	Total FOSs (g/L)	Purity	Recovery (%)	L*	a*	b*
Diluted crude FOSs	49.5 ± 2.2	–	100.0	76.96 ± 0.21	11.83 ± 0.07	12.55 ± 0.08
Biological treatment	41.6 ± 0.5	96.7	84.0	81.16 ± 0.19	8.57 ± 0.07	4.87 ± 0.04
Chromatography	38.6 ± 2.1	100.0	78.0	82.05 ± 1.80	− 2.27 ± 0.06	12.12 ± 0.51
Refined sugar	–		–	85.81 ± 0.21	− 0.65 ± 0.02	2.73 ± 0.04

## Discussion

With regard to the FOSs yield, the specific total contents of kestose, nystose, and fructofuranosyl nystose (4.76 g/100 g fresh weight) present in the red onions were higher than those that were found in chicory (0.39 g/100 g fresh weight), garlic (1.20 g/100 g fresh weight), and white onions (2.01 g/100 g fresh weight) [19] but were less than those that were found in different varieties of onions including Sturon (14.08 g/100 g fresh weight), Hysam (13.87 g/100 g fresh weight), Durco (9.68 g/100 g fresh weight), Grano de Oro (2.33 g/100 g fresh weight), and Caribo (10.35 g/100 g fresh weight) [20]. Notably, red onions contained high amounts of neokestose, which accounted for 1.92 g/100 g fresh weight, thus elevating the FOSs content up to 6.68 g/100 g of fresh weight. Still, comparisons of the yields of individual FOSs obtained from plant sources, specifically onions, were difficult since there are many forms of FOSs that depend upon the type and variety of onion [21]. This would be related to the complexed enzymatic biosynthesis of FOSs, which in turn limited the quantitative detection of FOSs. On the other hand, it is inevitable to state that onions contain high fructose, glucose, and sucrose contents [20, 21], which is in agreement with the results of this study. Prior to using red onion extract as an effective FOS prebiotic, it would be necessary to remove these sugars from the extract.

This research study attempted to screen selected yeast species that were able to efficiently remove the unwanted sugars present in FOSs extracted from red onions. All yeast species were isolated from Miang, the fermented tea leaves of northern ethnic people of Thailand, and the flowers of Assam tea collected near the Miang plantation areas. Current research has found that the diversity of the yeast species of Miang were associated with the yeast communities present in the flowers of the Assam tea [17, 18]. Nectar consists of sucrose as the main sugar, followed by glucose and fructose, which are varied depending upon the flower type. Nectar is an important energy source for visitors and pollinators [22, 23]. It has been noticed that the sugar composition of the nectar was similar to the substances involved with FOS synthesis. Therefore, it is highly possible that the yeast strains isolated from Miang and Assam flowers would be able to selectively utilize the unwanted sugars in the FOSs extracted from red onions.

The bottle neck of FOSs purification by microbial treatment is that yeast can only partially remove sucrose from crude FOSs. Any addition of exogeneous β-fructosidase could be evidence of an alternative method for the removal of sucrose. It could then be viewed as a key enzyme that is involved in two different reactions. First, β-fructosidase hydrolyzes sucrose to fructose and sucrose, which is then utilized by yeast [13]. It has been noted that the purification of FOSs by this strategy must be implemented carefully since treatment with high hydrolytic activity of the enzyme causes degradation of the produced FOSs. Second, β-fructosidase catalyzes transfructosylation of fructose to sucrose, while short chain FOSs are also formed. In the presence of high sucrose and fructose concentrations and with a low level of glucose, the transfructosylation activity of β-fructosidase was enhanced [24]. Yet, this strategy has led to changes in the type and quantity of FOSs that have been previously produced [4].

Unlike simple sugars, like glucose and fructose, yeast metabolites sucrose via hydrolysis of sucrose to fructose and glucose by using invertase or β-fructosidase, which was driven by the *Suc2* gene. Yeast strains that have insufficient β-fructosidase can be employed in the assimilation of sucrose via broad-substrate range α-glucosidase/H^+^ symporters and α-glucosidase. By this mechanism, their sucrose metabolisms are at a low level [25]. Yeast strains that have neither one of the previously mentioned mechanisms, are unable to ferment or assimilate sucrose. Yeast selectively utilizes kestose rather than other forms of FOSs. In these circumstances, these yeast strains may be able to produce a specific β-fructosidase that has high specificity in a limited substrate (kestose), which also has occurred in *Lactobacillus* spp. and *Bacteroide* sp. [26, 27]. Biochemical characteristics for sugar assimilation and fermentation of all tested yeast strains correspond to the species characteristics of yeast taxonomy [28]. Efficient utilization of FOSs can be focused on the hydrolytic activity of inulolytic enzymes including endo-inulinase and β-fructosidase. There is evidence indicating that high level secretions of β-fructosidase are also key factors in complete hydrolysis of inulin, as has been reported in the case of *S. cerevisiae* [11]. Moreover, the utilization of FOSs requires the activity of multiple operons associated with sugar metabolisms. The inactivity or absence of these operons may limit degradation of FOSs [29], which partially corresponds to the inability to utilize FOS of *C*. *orthopsilosis* FLA44.2 and *P. melissophilus* FLA48. Taking into consideration that extracellular β-fructosidase of *C*. *orthopsilosis* FLA44.2 and *P*. *melissophilus* FLA48 should have similar mechanisms for the purification of crude FOSs, the removal efficiency has proved that it is dependent upon the growth of these yeast strains. Growth of *P*. *melissophilus* FLA48 was partially inhibited as it exhibited a long lag phase when grown at high carbohydrate concentrations. Nonetheless, these yeast strains grew in crude FOSs without supplementation of additional nutrients. It is believed that extracellular β-fructosidase of the yeast strains may exhibit hydrolytic activity towards sucrose rather than transfructosylation activity as opposed to those included in the previous reports [4]. Accordingly, no changes in the quantities of neokestose, kestose, nystose, and fructofuranosyl nystose were relatively corresponded with the removed sucrose. To our knowledge, β-fructosidase is unlikely able to produce FOSs during purification since the enzyme activity was found at low levels after FOSs purity had been significantly increased by up to 95%, It was later determined that glucose, the inhibitor of the β-fructosidase, and fructose were completely removed. The presence of cell bound β-fructosidase throughout the cultivation of the yeast strains in the crude FOSs seems to have no effect on the removal of sucrose, but it is supposed that this enzyme could react with other FOSs, leading to decreased FOSs recovery.

Although the recovered FOSs require further purification steps, such as deproteinization, deionization, decolorization, and deodorization, one of the import concerns is the biological safety of *C*. *orthopsilosis* FLA44.2 and *P*.* melissophilus* FLA48. Hemolysin is a class of exotoxin proteins that have the ability to lyse red blood cells as well as nucleated cells. Hemolysin can be produced by several microorganisms and certain yeast strains including *C*. *albicans*, *C*. *glabarata*, and *Cryptococcus neoformans* [30]. Currently, there is no record of the production of hemolysin from *P*. *melissophilus*. As opposed to *C*. *orthopsilosis*, this yeast strain is a member of the *C*. *parapsilosis* complex and is classified as an opportunistic pathogen. It can be found in the blood, nails, urine, catheters, and virginal swabs [31], although it occurs widely in nature including in fruits and vegetables [32]. However, virulence between the clinical and non-clinical strains has not yet been investigated. Recently, a comparative study on the virulence characteristics of endophytic yeast strains of opportunistic *C*. *parapsilosis* isolated from fruit and vegetable products, and a study involving a clinical strain as the control, has been conducted via analysis of ability to produce active hydrolytic enzymes such as phospholipase, protease, and hemolysin. The results indicate that there are some isolates that exhibited less virulence than the control [33]. Preliminarily, the results showed that *C*. *orthopsilosis* FLA44.2 and *P*. *melissophilus* FLA48 were safe to use in food processing based on γ-hemolysin production, while they exhibited no cytotoxic effects on the viability of the human intestinal epithelial cell line; nevertheless, further safety assessments will be required. Furthermore, biosafety concerns are highly recommended during their use in the purification of FOSs.

The activated charcoal column was used for decolorization and deodorization of the purified FOSs obtained from yeast cultivation. Purified FOSs powder appeared as a white granular substance with an acceptable color that was similar to that of refined sugar. Onions possess a variety of sulfur-containing volatile compounds, including dimethyl trisulfide, dipropyl trisulfide, and dipropyl disulfide, which contribute to their unique and unpleasant characteristics [34]. That determination agrees with the results of this study in which dimethyl disulfide and dipropyl trisulfide were completely removed after the purified FOSs obtained from *P*. *melissophilus* FLA48 culture had passed through the activated charcoal column. Even after becoming odorless, some amount of dipropyl trisulfide residue remained in the powder, which was produced from a culture of *C*. *orthopsilosis* FLA44.2. However, the dimethyl disulfide was completely displaced after the same purification procedure. Thus, it is essential to optimize the ratio between the purified FOSs obtained from the yeast treatment and the activated charcoal. Furthermore, the purified FOSs have been confirmed to have no effect on cell viability of the small intestinal epithelial cell line (data not shown).

As far as all of the published reports are concerned, many physicochemical and microbial treatment strategies have been employed for the purification of FOSs produced by enzymatic and/or microbial transformation as opposed to those that were directly extracted from plants. The current research on the purification of FOSs extracted from garlic has shown that activated charcoal could recover FOSs by 88.99% with the highest purity of 94% after elution with 15% ethanol [10]. Our results have shown that the combination of yeast treatment and activated charcoal led to 78.0 to 82.8% recovery with no fructose, glucose, or sucrose being detected. Importantly, the results provide new insight into an alternative process that could effectively purify FOSs from plant sources. In addition, *C. orthopsilosis* and *P. melissophilus* may be applied for the purification of FOSs produced by enzymatic and/or microbial transformation.

## Conclusion

The study has reported on the screening of yeast strains that are capable of selectively removing unwanted sugars, namely fructose, glucose, and sucrose, for the purification of FOSs extracted from red onions. Among the 43 yeast species isolated from Miang and the Assam tea flowers, only *C*. *orthopsilosis* and *P*. *melissophilus* exhibited potential in the removal of unwanted sugars from crude FOSs without the addition of other medium compositions. Both strains have displayed feasible potential for purification of FOSs, while FOSs that were extracted from plants were highly recommended. Being more efficient, the ability to directly purify the original concentration of crude FOSs, *C*. *orthopsilosis* exhibited a high specific growth rate, as well as higher percentages of FOS purity and recovery than *P*. *melissophilus*. Importantly, the current results in terms of hemolysin production and cell cytotoxicity indicated that these yeast strains are safe for use in food processing; however, further assessment would be essential to explicitly assure safety prior to being applied in the large-scale purification of FOS. Nevertheless, this study has offered activated charcoal as a tool for decolorization, deodorization, and detoxification in cases where toxins are generated by the microorganisms used for the purification of FOSs. The final purified FOSs was prepared as an odorless and white granular powder that was similar to that of refined sugar. Overall, the presented purification process provides new insight into the utilization of alternative yeast species for the purification of FOSs.

## Materials and methods

### Raw materials, chemicals, and culture media

Red onions were purchased from the Muang Mai Market (18.797334, 98.9951373). High performance liquid chromatography (HPLC) standard 1-kestose, nystose, and fructofuranosyl nystose were purchased from Fujifilm Wako Chemical Co. (Osaka, Japan), while analytical standard grade glucose, fructose, and sucrose were obtained from Sigma-Aldrich (St. Louis, MO, USA). Neokestose was prepared according to the modified method described in the previous study [35]. Small intestine HIEC-6 (ATCC® CRL-3266™) was obtained from the American Type Culture Collection (ATCC) (Manassas, VA, USA). Materials for cell cultures and cell cytotoxicity tests, such as Opti-MEM-reduced serum medium, HEPES, GlutaMAX, epidermal growth factor, fetal bovine serum, 3-(4,5-dimethylthiazol-2-yl)-2,5-diphenyltetrazolium bromide solution (MTT), and dimethyl sulfoxide (DMSO), were of the highest available quality and were obtained from ThermoFisher Scientific Inc. (Waltham, MA, USA). The microbial culture medium ingredients used in this study, such as yeast extract, malt extract, and agar, were all purchased from HiMedia (Nashik, India). Sheep’s blood agar was derived from M&P Impex (Bangkok, Thailand). Amberlite® MB20 mixed ion exchange resin and activated charcoal were obtained from Sigma Aldrich (Darmstadt, Germany).

### Microorganisms, culture conditions, and inoculum preparation

A total of 43 different yeasts species were used in this study and were isolated from Miang and the flowers of *Cm*. (*Camellia) sinensis* var. *assamica*, as has been indicated in previous studies [17, 18]. They were stored at −20 °C in yeast extract-malt extract broth (YMB) supplemented with 15% (v/v) glycerol. When necessary, each species was cultivated in YMB on a 150-rpm rotary shaking incubator at 30 °C for 24–48 h. To isolate a single colony of yeast, a loopful of the prepared culture was streaked on yeast extract-malt extract agar (YMA) and incubated at 30 °C for 48 h. For inoculum preparation, a single colony of yeast was inoculated in a 125-mL Erlenmeyer flask containing 50 mL of YMB (with a working volume of 40%) and incubated under the same conditions described above or until the culture reached a maximum optical density of 600 nm (OD_600_) of 8–9.

### Extraction of fructooligosaccharides from red onions

The outer layer of the fresh red onions was removed by cutting the top and bottom sections of the onions. The peeled red onions were then chopped into small cubes, homogenized, and extracted using an JT-2010 Healthy Slow Juicer (Jutian, China). The red onion extract was placed on a hot plate stirrer at 80 °C for 30 min, left to cool, and centrifuged at 40,000 × g for 10 min to inactivate the endogenous enzymes present in the red onions and to remove any insoluble fractions. The clear supernatant was assigned as crude fructooligosaccharides (FOSs). This solution was used for proximate analysis and to determine total carbohydrate (TC) content using the phenol–sulfuric acid method, sugars and oligosaccharide contents by employing the high-performance liquid chromatography (HPLC) technique.

### Screening of yeast strains for selective removal of glucose, fructose, and sucrose from crude FOSs

The crude FOSs extracted from red onions was diluted with distilled water to yield a two-fold dilution. The diluted crude FOSs were passed through a 0.22 μm membrane filter to achieve a sterile solution and used as culture medium in further experiments. A total volume of 1 mL of yeast inoculum was transferred to a 1.5 mL microtube and centrifuged at 17,350 × g for 2 min. The supernatant was removed and the cell pellet was resuspended with 1 mL of 0.85% (w/v) NaCl solution. The suspension was transferred into a 40 mL screw cap tube containing 20 mL of the diluted crude FOSs. The resulting cell suspension was cultivated on a 150-rpm rotary shaker at 30 °C for 96 h. Samples were periodically collected every 24 h of the cultivation process, heated at 90 °C for 10 min to inactivate yeast viability and that of any enzymes, left to cool, and centrifuged at 17,350 × g for 5 min. The clear supernatant was then used for detection of residual glucose, fructose, and sucrose by thin-layer chromatography (TLC). Yeast strains that were able to specifically remove fructose, glucose, and sucrose with minimal effect on FOSs content were presumptively selected for further characterization.

### Confirmation of selected yeast strains

In our confirmation step, the experiment was conducted as has been previously described. Samples were periodically taken at 0, 24, 48, 72, and 96 h of cultivation. A portion of the collected samples was heated at 90 °C for 10 min prior to centrifugation. The clear supernatant was then used for determination of residual fructose, glucose, sucrose, and FOSs by HPLC and determination of TC content. The purity of the glucose, fructose, and sucrose obtained from crude FOSs was calculated according to the following equation:

$$\% {\text{ Purity}} = \left[ {1 - \frac{{{\text{Residual FGS}}\left( {{{\text{g}} \mathord{\left/ {\vphantom {{\text{g}} {\text{L}}}} \right. \kern-0pt} {\text{L}}}} \right)}}{{{\text{Initial FGS }}\,\left( {{{\text{g}} \mathord{\left/ {\vphantom {{\text{g}} {\text{L}}}} \right. \kern-0pt} {\text{L}}}} \right)}}} \right]\, \times \,{100}$$

The percentage recovery of the retained FOSs was calculated according to the equation shown below:

$$\% {\text{ Recovery }} = \frac{{{\text{Retained}} {\text{ FOSs}}\,\left( {{{\text{g}} \mathord{\left/ {\vphantom {{\text{g}} {\text{L}}}} \right. \kern-0pt} {\text{L}}}} \right)}}{{{\text{Initial}} {\text{ FOSs}}\,\left( {{{\text{g}} \mathord{\left/ {\vphantom {{\text{g}} {\text{L}}}} \right. \kern-0pt} {\text{L}}}} \right)}} \times 100$$

Accordingly, the retained FOSs content was calculated from the difference between the retained TC and the retained total contents of fructose, glucose, and sucrose (FGS). Similarly, the initial FOSs content was calculated from the difference between the initial TC content of the crude FOSs and the initial content of FGS. Yeast strains that were able to specifically remove fructose, glucose, and sucrose with the least effect on recovery of FOSs were selected for further experimentation.

The rest of the collected samples were investigated for β-fructosidase activity. To prepare the enzyme fraction, the collected samples were centrifuged at 17,350 × g and 4 °C for 5 min. The clear supernatant was then assigned as the crude extracellular enzyme. Cell pellets were washed twice with 50 mM sodium phosphate buffer at pH 6.5 and then resuspended in the same buffer. The resulting cell suspension was assigned as the cell-bound enzyme. The β-fructosidase activity was assayed according to the method employed in a previous study [36] with some modifications. Briefly, β-fructosidase activity was assayed by mixing 50 μL of enzyme and 50 μL of 0.5% (w/v) sucrose prepared in 100 mM sodium phosphate buffer at a pH value of 6.5. The reaction was carried out at 30 °C for 30 min. Afterwards, the enzyme reaction was terminated by adding 100 μL of 3,5-dinitrosalicylic acid (DNS) reagent and heated at 100 °C for 10 min. After allowing the reaction to cool, 800 μL of distilled water was added and it was mixed. The absorbance was then measured at 540 nm. One unit of β-fructosidase was defined as the amount of enzyme that catalyzes the hydrolysis of sucrose to liberate the reducing sugars equivalent to glucose in 1 min under the assay conditions (0.5% sucrose (w/v), pH 6.5, 30 min reaction time). In this experiment, baker’s yeast of *S*. *cerevisiae* was cultured in the crude FOSs under the same conditions, as has been described above. Accordingly, this was determined to be a positive strain for the detection of β-fructosidase activity.

### Safety assessment of selected yeast strains

#### Hemolytic activity test

Hemolytic assay was conducted using sheep’s blood agar. Yeast strains were streaked on a blood agar plate and incubated at 30 °C for 72 h. *Staphylococcus aureus* TISTR 746 was used as the positive control. *Saccharomyces cerevisiae* isolated from baker’s yeast, which is considered a food grade yeast strain, was used as the negative control. Colonies that formed a clear zone and that were surrounded with a green hue were identified as β-hemolysis and α-hemolysis, respectively, while those that displayed no clear zone were identified as γ-hemolysis.

#### Cell cytotoxicity test

Human intestinal epithelial cells were cultivated in 96-well plates containing 180, 190, and 195 μL of OpiMEM 1 reduced serum medium that was supplemented with 20 mM HEPES, 10 mM GlutaMAX, 10 ng/mL epidermal growth factor (EGF), and 4% fetal bovine serum (FSB) at 37 °C under 5% CO_2_ atmosphere for a certain amount of time to achieve 5 × 10^3^ cells/mL per well. The prepared cells were then treated with 20, 10, and 5 µL of the samples obtained from the confirmation step in respective order and incubated under the same conditions, as has been explained. Cell culture without the addition of a sample was employed as a control. After 48 h of cultivation, the culture medium was removed from the plates, and the cell lines were washed by 100 µL of PBS at pH 7.4 prior to the addition of 100 µL of 3-(4,5-dimethylthiazol-2-yl)-2,5- diphenyltetrazolium bromide solution (MTT). The plates were incubated at 37 °C in a 5% CO_2_ atmosphere for 4 h to allow for the formation of formazan crystals that were then dissolved with 100 µL of DMSO. Finally, the absorbance of resulting solution was measured at 550 (A_550_) and 620 nm (A_620_). The percentage of cell viability was then calculated using the following equation:

$$\% {\text{Cell viability}} = \frac{{\left( {{\text{A}}_{{550}} - {\text{ A}}_{{620}} } \right) _{{{\text{Control}}}} - \left( {{\text{A}}_{{550}} - {\text{A}}_{{620}} } \right)_{{{\text{Sample}}}} }}{{\left( {{\text{A}}_{{550}} - {\text{A}}_{{620}} } \right)_{{{\text{Control}}}} }}\times 100$$

### Evaluation of removal efficiency of selected yeast strains in shake flask scale

Two-fold diluted FOSs and that without dilution were prepared and used as two different cultivation media for evaluation of removal efficiency of the selected yeast strains. A total volume of 5 mL of inoculum was centrifuged at 7300 × g for 10 min. The clear supernatant was discarded, and the cell pellets were then resuspended with 0.85% (w/v) NaCl solution. The resulting suspension was transferred to 250 mL Erlenmeyer flasks containing 50 mL of the different media. Cultivation was carried out on a 150-rpm rotary shaker at 30 °C. Samples were periodically collected at 0, 12, 24, 36, 48, 72, and 96 h of cultivation for measurement of optical density at 600 nm (OD_600_). The specific growth rate was calculated according to the following equation:

$${\text{ln OD}}_{600,{\text{t}}} - {\text{ln OD}}_{{600,0}} = {\mu t}$$

The collected samples were heated at 90 °C for 10 min, left to cool, and centrifuged at 17,350 × g for 10 min. The supernatant was then evaluated for glucose, fructose, sucrose, and total carbohydrates (TC). The percentage purity and the percentage recovery of the purified FOSs were calculated. Based on percentage purity, percentage recovery, and specific growth rate of the selected yeasts strains, either two-fold diluted FOSs or that without dilution (original concentration of FOSs) was selected for FOS purification in a fermenter scale.

### Evaluation of removal efficiency of selected yeast strains in 1-Lfermenter scale

A 1-L stirred tank fermenter (B.E. Marubishi Co., Ltd., Tokyo, Japan) with 60% working volume of the crude FOSs was prepared. An inoculum of 60 mL was centrifuged at 7300 × g for 10 min to remove the supernatant. Cell pellets were resuspended in 60 mL of 0.85% (w/v) NaCl and the resulting suspension was transferred to the fermenter. Cultivation was carried out at 30 °C with an agitation speed of 250 rpm and an aeration rate of 0.2 vvm. No pH adjustment was required. Samples were periodically taken at 0, 12, 24, 36, 48, 72, and 96 h of cultivation for measurement of OD_600_ and pH values. A portion of the collected samples was heated at 90 °C for 10 min to inactivate any enzyme and yeast viability, left to cool, and centrifuged at 17,350 × g for 5 min. The clear supernatant was employed to determine glucose, fructose, and sucrose contents, as well as TC. Finally, the percentage purity and the percentage recovery values of the purified FOSs were calculated.

### Decolorization and deodorization of the purified FOSs

The culture supernatant obtained from the purification in the fermenter scale was centrifuged at 7300 × g for 10 min. The clear supernatant was then mixed with two volumes of ice-cold ethanol and left at − 20 °C for at least 2 h in order to precipitate any soluble proteins. Insoluble particles and precipitated proteins were removed by centrifugation and the clear supernatant was evaporated at 50 ºC using a rotary evaporator. The resulting solution (100 mL) was demineralized by Amberlite® MB20 mixed ion exchange resin (3 g) at 20 °C for 4 h prior to being filtered to collect the supernatant for further decolorization and deodorization by activated charcoal column. Activated charcoal (100 g) was stirred with 100 mL of 37% hydrochloric acid at 25 °C for 30 min. It was then neutralized with 1% (w/v) NaOH, rinsed, and suspended with distilled water. The slurry was passed through Whatman No. 1 filter paper to collect the reactivated charcoal, which was then dried at 105 °C until it reached a constant weight. To prepare the activated charcoal column, 10 g of reactivated charcoal was suspended in 100 mL of deionized water and autoclaved to remove any air from the pores at 121 °C, 15 lb/in^2^ for 15 min. All charcoal suspensions were loaded into a glass column (3.5 × 30 cm) that was equipped with a Millipore pump with a maximum flow rate. The system was then operated from the top to the bottom of the column. After column equilibration, 100 mL of demineralized FOSs solution was loaded into the column, then 100 mL of deionized water was applied to flush away any unbinding substances. The FOSs were eluted by 200 mL of 50% (v/v) ethanol with a flow rate of approximately 1 mL/min at the maximum flow rate of the pump. The eluted solution was centrifuged at 17,350 × g for 10 min and the clear supernatant was then evaporated at 50 °C using a rotary evaporator to achieve the eluted fraction. The crude FOSs, culture broth, and eluted fraction were evaluated for glucose, fructose, and sucrose contents, as well as TC content. Percentage recovery was then calculated by employing the previously described equation. In addition, the purified FOSs were freeze-dried into a powder. Crude FOSs and the culture broth of crude FOSs by each yeast species were also freeze dried. Each FOSs powder sample was identified by color differences using the L * a * b system. Finally, the cytotoxicity of the purified FOSs powder was determined in the human intestinal epithelial cells according to the method previously described. In addition, volatile compounds associated with the red onion extract were analyzed from both crude and purified FOSs using solid phase microextraction gas chromatography-mass spectrometry (SPME–GC–MS) (Additional file).

### Thin layer chromatography

Thin layer chromatography (TLC) was performed according to the method employed in a previous study [5]. In brief, 1 μL of the sample or standard was spotted on an aluminum silica gel plate. The spotted plate was developed in a mobile phase chamber containing n-butanol: ethanol: water in ratio of 5: 3: 2 (v/v/v). The developing procedure was conducted three times for optimal separation of monosaccharides, disaccharides, and FOSs. The sugars were then visualized by being sprayed with 0.5% (w/v) thymol in a mixed solution of 5% (v/v) H_2_SO_4_ prepared in ethanol. They were then heated at 100ºC in a hot air oven for 10 min.

### High performance liquid chromatography

Glucose, fructose, and sucrose contents were analyzed by high performance liquid chromatography (HPLC). Briefly, the HPLC system was connected to a Shodex Asahipak (NH_2_P-50 4E) column (Shodex, Tokyo, Japan) that was equilibrated with a mobile phase (acetonitrile: deionized water at a ratio of 75: 25 (v/v)). Separation was performed at 30 °C with a flow rate of 0.5 mL/min. The separated sugars were detected by refractive index (RI) detector. The specific FOSs, including kestose, neokestose, nystose, and fructofuranosyl nystose, were analyzed with use of a Shodex HILICpak VN-50 4D column that had been equilibrated with a mobile phase consisting of acetonitrile: deionized water at a ratio of 80: 20 (v/v). The separation conditions were set at 30 °C with a flow rate of 0.5 mL/min. The separated oligosaccharides were then detected using an RI detector.

### Total carbohydrates

Total carbohydrates were determined by phenol–sulfuric acid method [37]. Briefly, 0.25 mL of the properly diluted sample and 0.25 mL of 5% (w/v) phenol solution were transferred to a glass test tube with a cap. Then, 1.25 mL of concentrated sulfuric acid was carefully transferred to the mixture and vortexed until homogeneity was achieved. The reaction was allowed to stand at room temperature (25 °C) for 30 min prior to measuring the absorbance at 490 nm. Fructose was then used to create a standard curve.

### Proximate analysis

Moisture, crude fat, protein, fiber, ash, and carbohydrate contents were quantified according to the methods of AOAC (2012). A nitrogen-to-protein conversion factor of 6.25 was used to calculate the concentrations of the proteins that were present in the samples.

### Color analysis

Evaluation of surface color was performed using a portable tristimulus colorimeter (Minolta Chroma Meter CR-300, Osaka, Japan). The results were presented by employing the L * a * b color coordination system, where L* represents lightness, a* is red ness, and b* represents yellowness.

### Supplementary Information

**Additional file 1: Figure S1.** FOS profile obtained from cultivation of different yeast strains in crude FOSs extracted from red onions. **Figure S2.** Appearance of FOS powder obtained from each step of purification. **Figure S3.** Calibration curves obtained by plotting the average of mean peak areas and concentrations of sugars and FOSs. **Table S1.** Classification of yeast species isolated from Miang and Tea flower based on fructose, glucose, sucrose, and FOS utilizations. **Table S2.** Retention time, slope and R^2^ value for analysis of sugars and FOSs extracted from red onion. **Table S3.** Selected volatile compounds from crude FOSs and purified FOSs.

## Data Availability

Not applicable.
