# Supplementary material for: Purification and characterization of crude fructooligosaccharides extracted from red onion (Allium cepa var. viviparum) by yeast treatment
Source: Microb Cell Fact. 2024 Jan 10;23:17. doi: 10.1186/s12934-023-02289-7 (PMC10782719; doi:10.1186/s12934-023-02289-7)
Supplement: Supplementary file 1 — Additional file 1: Figure S1. FOS profile obtained from cultivation of different yeast strains in crude FOSs extracted from red onions. Figure S2. Appearance of FOS powder obtained from each step of purification. Figure S3. Calibration curves obtained by plotting the average of mean peak areas and concentrations of sugars and FOSs. Table S1. Classification of yeast species isolated from Miang and Tea flower based on fructose, glucose, sucrose, and FOS utilizations. Table S2. Retention time, slope and R2 value for analysis of sugars and FOSs extracted from red onion. Table S3. Selected volatile compounds from crude FOSs and purified FOSs. [file 12934_2023_2289_MOESM1_ESM.docx]

**Supplementary Material**

**Purification and characterization of crude fructooligosaccharides extracted from red onion (*Allium cepa* var. *viviparum*) by yeast treatment**

Jakkrit Aisara^1,2†^, Jirat Wongsanittayarak^1,2†^, Nalapat Leangnim^2,3^, Kraikrit Utama^2,3^, Padchanee Sangthong^2^, Woraprapa Sriyotai^4^, Sugunya Mahatheeranont^4^, Suphat Phongthai^5^, Kridsada Unban^5^, Saisamorn Lumyong^6^, Chartchai Khanongnuch^7^, Pairote Wongputtisin^8^, Apinun Kanpiengjai^2*^

^1^ Program in Biotechnology, Multidisciplinary and Interdisciplinary School, Chiang Mai University, Chiang Mai 50200, Thailand

^2^ Division of Biochemistry and Biochemical Innovation, Department of Chemistry, Faculty of Science, Chiang Mai University, Chiang Mai 50200, Thailand

^3^ Office of Research Administration, Chiang Mai University, Chaing Mai 50200, Thailand

^4^ Department of Chemistry, Faculty of Science, Chiang Mai University, Chiang Mai 50200, Thailand

^5^ Division of Food Science and Technology, School of Agro-Industry, Faculty of Agro-Industry, Chiang Mai University, Chiang Mai 50100, Thailand

^6^ Division of Microbiology, Department of Biology, Faculty of Science, Chiang Mai University, Chiang Mai, 50200, Thailand

^7^ Department of Biology, Faculty of Science, Chiang Mai University, Chiang Mai, 50200, Thailand

^8^ Program in Biotechnology, Faculty of Science, Maejo University, Chiang Mai 50290, Thailand

^9^ Academy of Science, The Royal Society of Thailand, Bangkok 10300, Thailand

^†^Jakkrit Aisara and Jirat Wongsanittayarak contributed equally to this work and share first authorship.

*Corresponding author: Apinun Kanpiengjai

Corresponding author’s e-mail: ak.apinun@gmail.com, apinun.k@cmu.ac.th

**Fig. S1** FOS profile obtained from cultivation of different yeast strains in crude FOSs extracted from red onions

**Fig. S1** FOS profile obtained from cultivation of different yeast strains in crude FOSs extracted from red onions (continued)

**Fig. S1** FOS profile obtained from cultivation of different yeast strains in crude FOSs extracted from red onions (continued)

**Fig. S1** FOS profile obtained from cultivation of different yeast strains in crude FOSs extracted from red onions (continued)

**Fig. S1** FOS profile obtained from cultivation of different yeast strains in crude FOSs extracted from red onions (continued)

| Yeast | Crude FOSs | Yeast treatment | Activated charcoal |
| --- | --- | --- | --- |
| *Candida orthopsilosis* FLA44.2 | 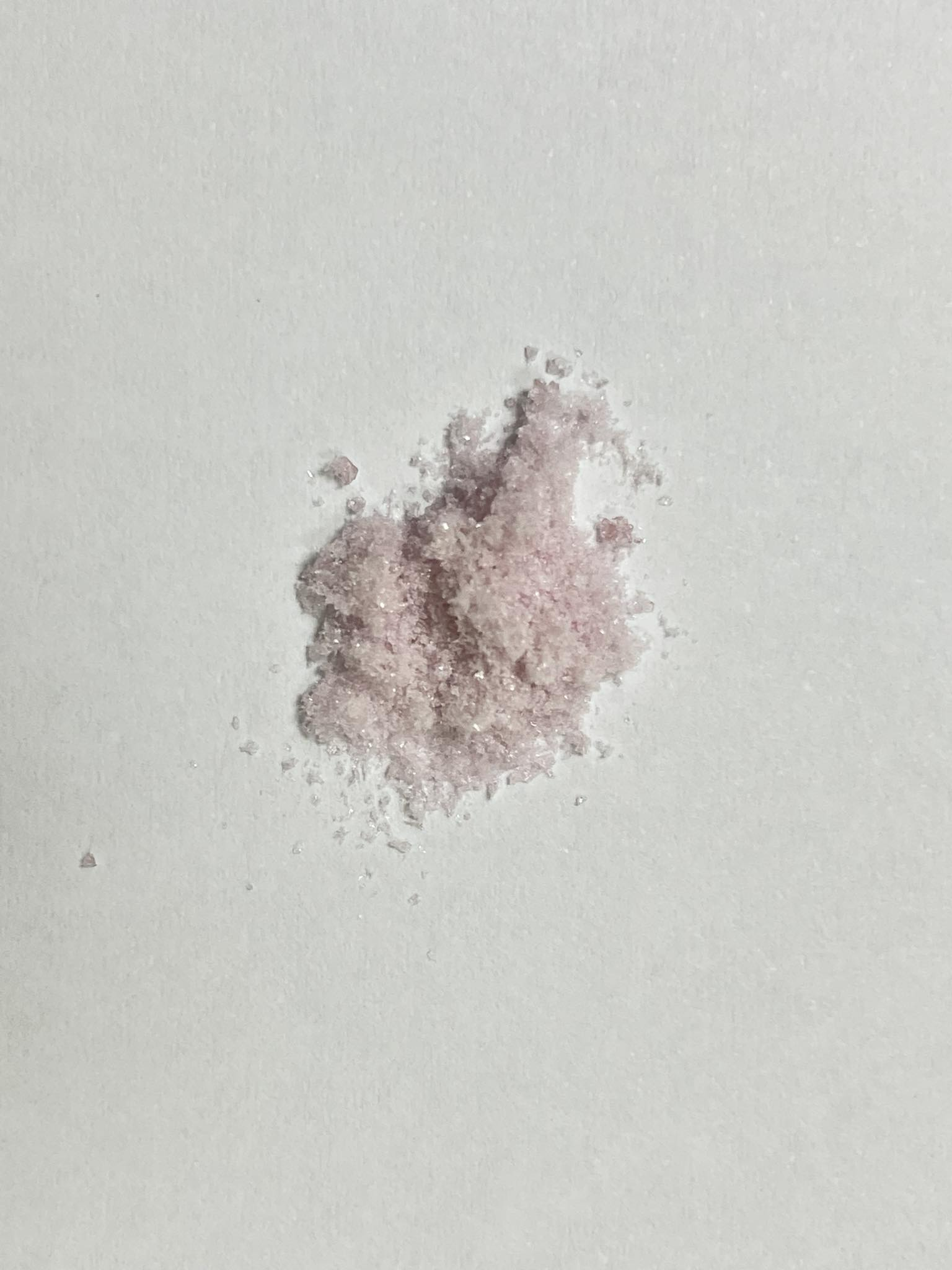 | 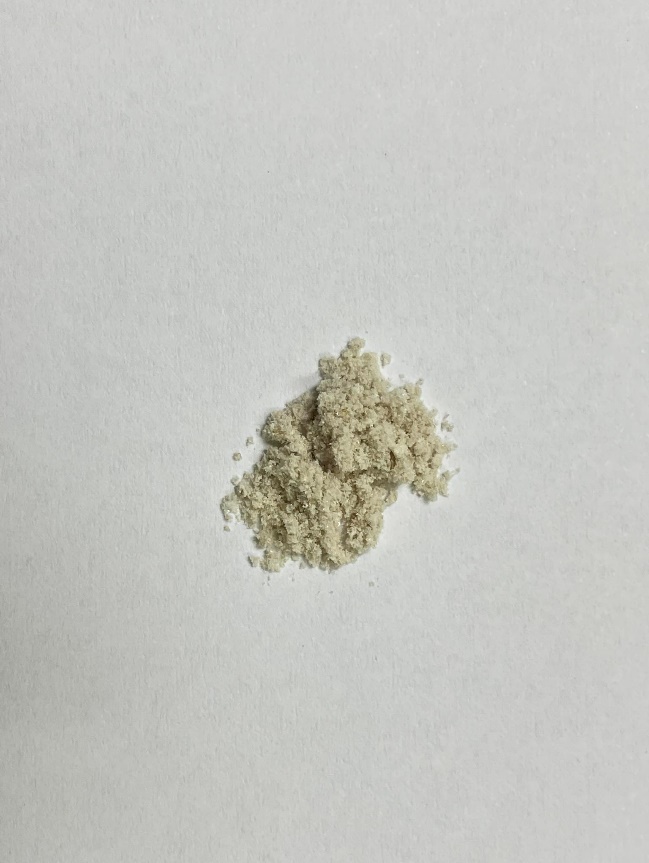 | 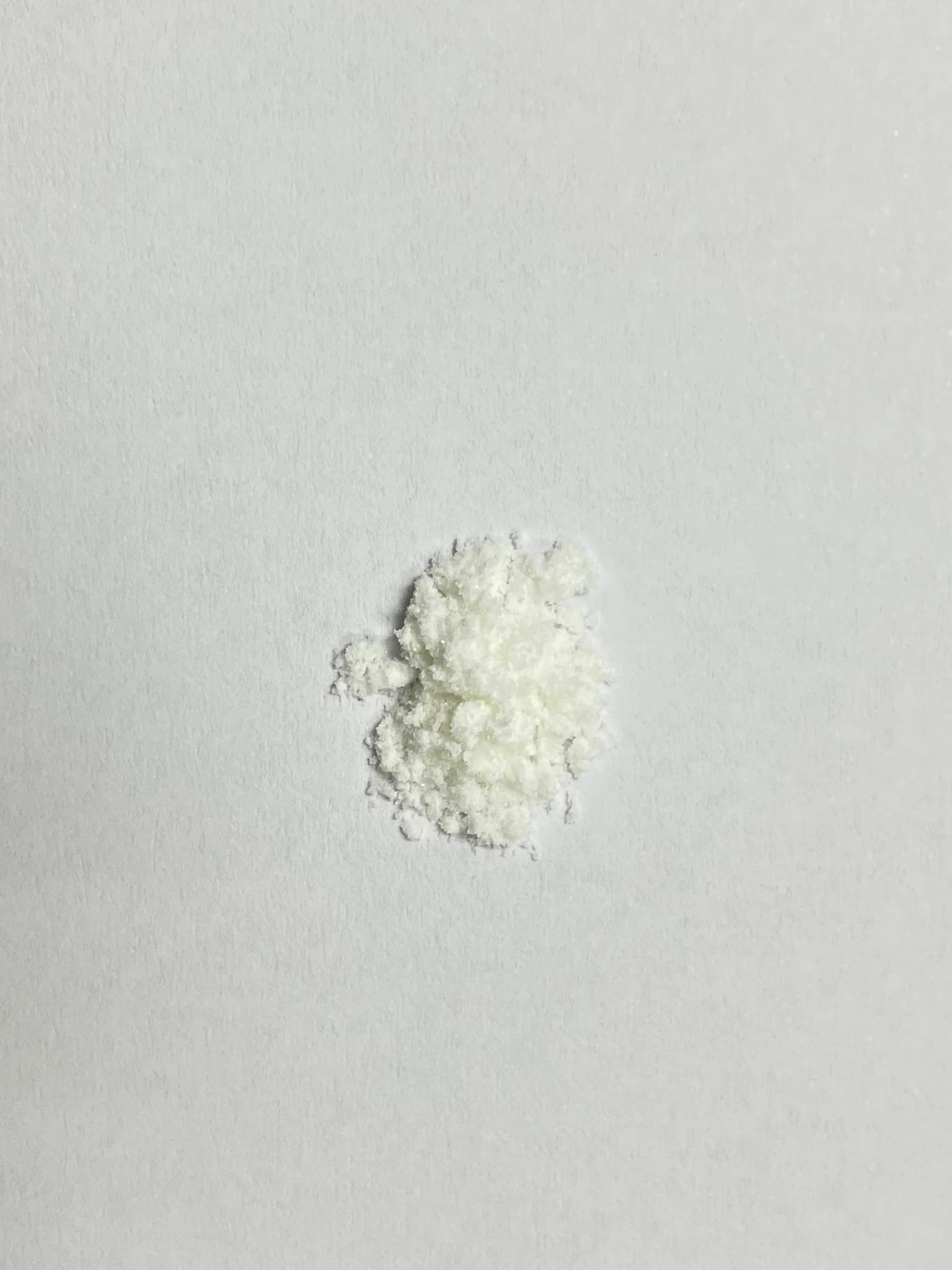 |
| Yeast | 2×diluted crude FOSs | Yeast treatment | Activated charcoal |
| *Priceomyces melissophilus* FLA48 | 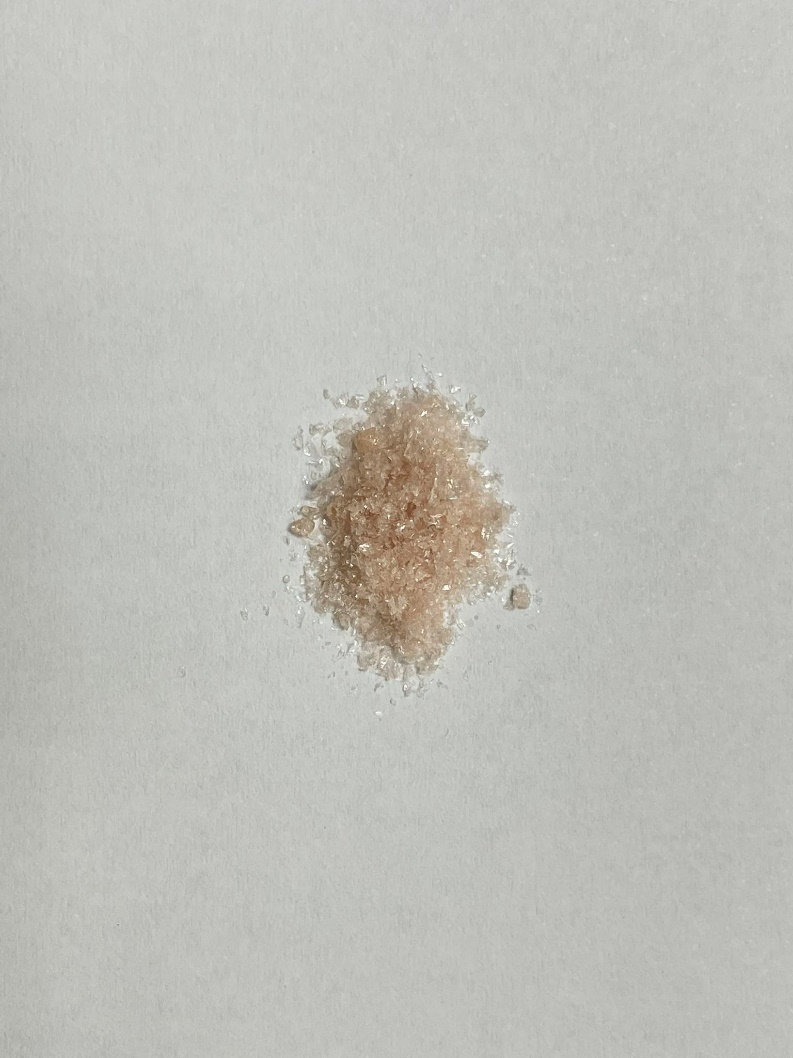 | 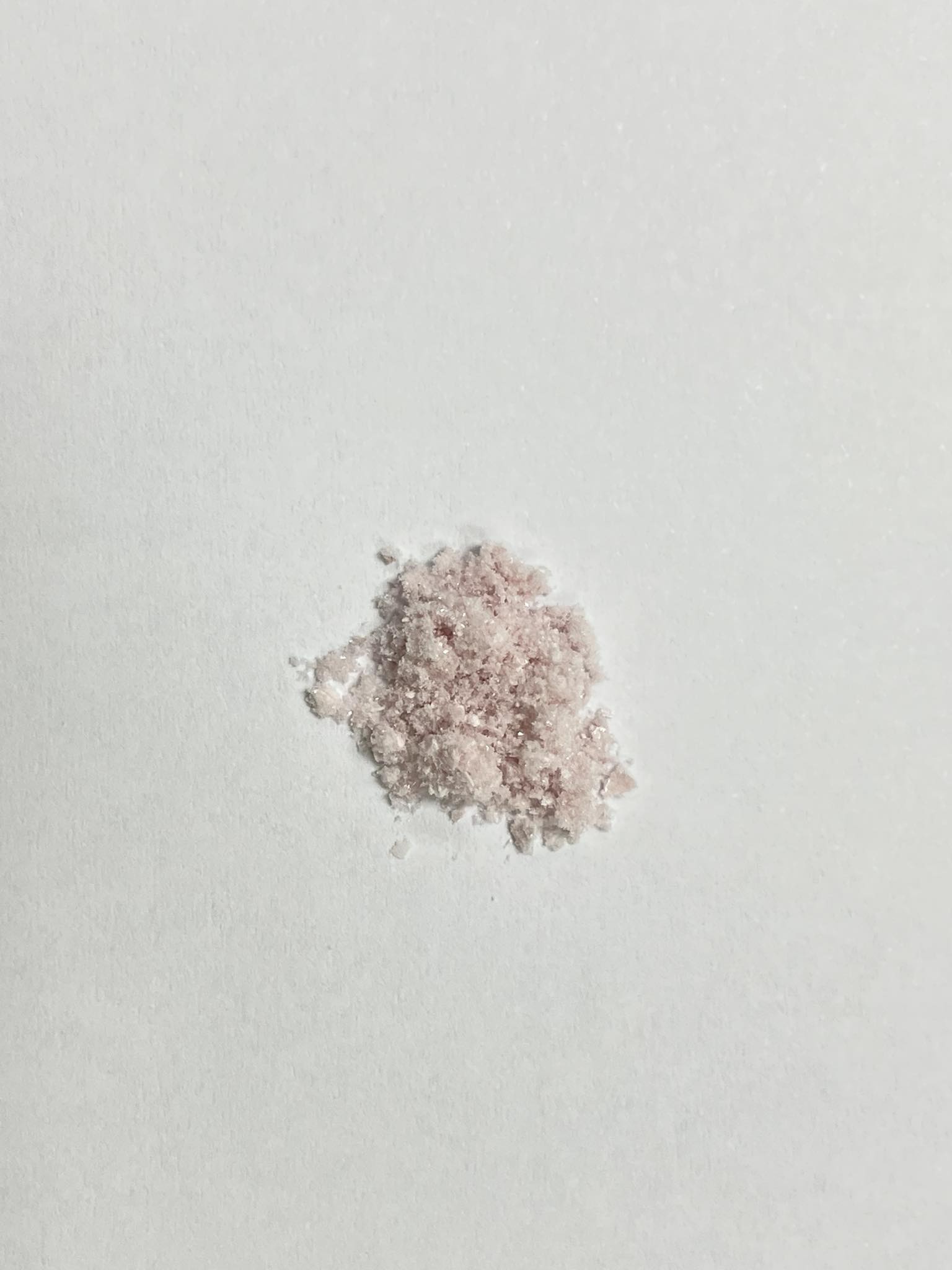 | 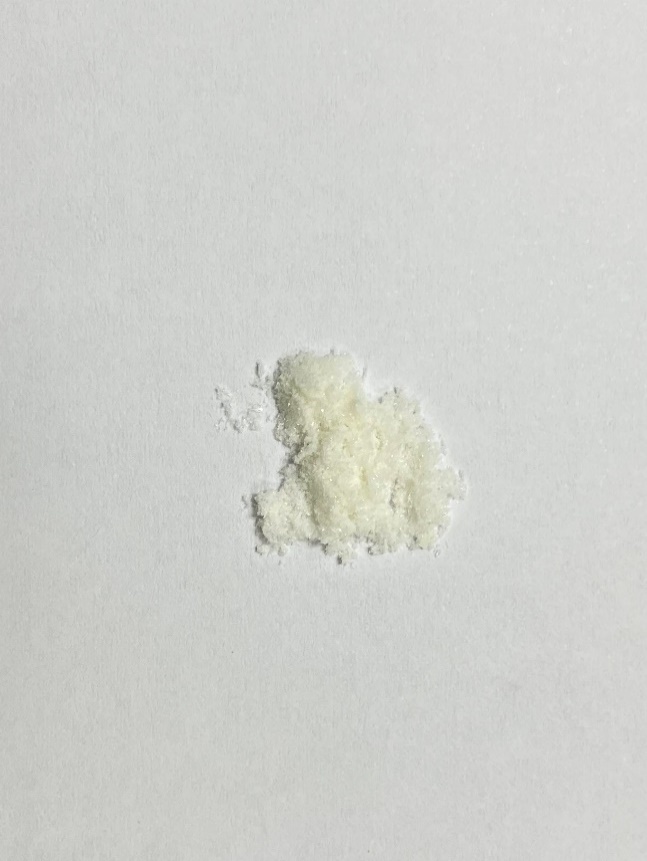 |

**Fig. S2** Appearance of FOS powder obtained from each step of purification

**Table S1** Classification of yeast species isolated from Miang and Tea flower based on fructose, glucose, sucrose, and FOS utilizations

| **Group**  **(no. of species)** | **Species** | **Strains** | **Source** | **Fermentation of sucrose*** | **Assimilation of sucrose, inulin*** |
| --- | --- | --- | --- | --- | --- |
| 1  (19) | *Candida boidinii* | A9.2 | Miang | - | v,- |
|  | *Candida bombi* | FLA45.2 | Tea flower | + | +,- |
|  | *Candida chrysomelidarum* | FLA22.1 | Tea flower | - | +,- |
|  | *Candida drosophilae* | FLA11.5 | Tea flower | - | - |
|  | *Candida ethanolica* | A10.2 | Miang | - | - |
|  | *Candida hawaiiana* | FLA6.1 | Tea flower | n | +,- |
|  | *Candida stellimalicola* | A32.5 | Miang | n | - |
|  | *Candida xylopsoci* | FLA44.1 | Tea flower | - | - |
|  | *Diutina catenulata* | FLA33 | Tea flower | - | - |
|  | *Hanseniaspora opuntiae* | FLA9.2 | Tea flower | + | +,- |
|  | *Hanseniaspora thailandica* | FLA55.2 | Tea flower | n | - |
|  | *Metschnikowia koreensis* | FLA1.2 | Tea flower | - | +,- |
|  | *Metschnikowia laotica* | FLA31.2 | Tea flower | n | +,- |
|  | *Pichia kluyveri* | FLA9.3 | Tea flower | - | - |
|  | *Pichia kudriavzevii* | A14.1 | Miang | - | - |
|  | *Pichia manshurica* | A2.2 | Miang | - | - |
|  | *Pichia occidentalis* | A6.1 | Miang | - | - |
|  | *Saccharomycopsis fodiens* | FLA17.3 | Tea flower | n | - |
|  | *Wickerhamiella dulcicola* | FLA5.4 | Tea flower | n | +,- |
| 2  (7) | *Candida azyma* | FLA1.1 | Tea flower | - | +,- |
|  | *Candida quercitrusa* | FLA11.3 | Tea flower | v | +,- |
|  | *Cyberlindnera rhodanensis* | A45.3 | Miang | v | +,- |
|  | *Debaryomyces hansenii* | A28.2 | Miang | v | +, v |
|  | *Hannaella pagnoccae* | FLA26.1 | Tea flower | n | +,v |
|  | *Nakazawaea holstii* | FLA11.4 | Tea flower | - | +,- |
|  | *Yamadazyma terventina* | FLA47.2 | Tea flower | n | +,- |

**Table S1** Classification of yeast species isolated from Miang and Tea flower based on fructose, glucose, sucrose, and FOS assimilations (continued)

| **Group** | **Species** | **Strains** | **Source** | **Fermentation of sucrose*** | **Assimilation of sucrose, inulin*** |
| --- | --- | --- | --- | --- | --- |
| 3  (15) | *Candida leandrae* | FLA4.1 | Tea flower | n | +,+ |
|  | *Candida pseudohaemulonii* | FLA40.1 | Tea flower | n | +,- |
|  | *Cyberlindnera fabianii* | FLA39.2 | Tea flower | + | +,- |
|  | *Debraryomyces nepalensis* | FLA31.1 | Tea flower | v | +,- |
|  | *Hyphopichia burtonii* | A39.5 | Miang | + | +,- |
|  | *Kodamaea nitidulidarum* | FLA11.1 | Tea flower | n | +,- |
|  | *Kodamaea ohmeri* | FLA31.4 | Tea flower | + | +,v |
|  | *Meyerozyma caribbica* | FLA47.1 | Tea flower | + | +,+ |
|  | *Moesziomyces antarcticus* | FLA50 | Tea flower | n | +,- |
|  | *Papiliotrema flavescens* | FLA31.5 | Tea flower | - | +,- |
|  | *Papiliotrema laurentii* | FLA51.1 | Tea flower | - | +,- |
|  | *Pseudozyma hubeiensis* | FLA3.1 | Tea flower | n | +,- |
|  | *Sporidiobolus ruineniae* | A45.2 | Miang | - | +,- |
|  | *Wickerhamyces anomalus* | FLA39.1 | Tea flower | + | +,- |
|  | *Wickerhomyces ciferrii* | FLA11.2 | Tea flower | + | +,- |
| 4  (2) | *Candida orthopsilosis* | FLA44.2 | Tea flower | - | +,- |
|  | *Priceomyces melissophilus* | FLA48 | Tea flower | - | +,+ |

**Note:** Group 1 Fructose and glucose were assimilated.

Group 2 Fructose, glucose, and kestose were assimilated.

Group 3 FOSs were assimilated.

Group 4 Fructose, glucose, and sucrose were assimilated.

* Data collected from Kurtzman, C.P. (2011)

**Reference:** Kurtzman CP, Fell JW, Boekhout T: Summary of species characteristics. In *The Yeasts (Fifth Edition).* Edited by Kurtzman CP, Fell JW, Boekhout T. London: Elsevier; 2011: 223-277.

**Analysis of volatile compounds using solid phase microextraction gas chromatography-mass spectrometry (SPME-GC-MS)**

The volatile compounds in the sample were determined by employing solid-phase microextraction gas chromatography-mass spectrometry. The 6890 gas chromatography device was equipped with a 5975B mass selective detector (GC–MS; Agilent Technologies, Santa Clara, CA, USA). The head space volatiles were analyzed using a SPME fiber coated with a 50/30 μm-thick layer of DVB/CAR/PDMS (Supelco Inc., Bellefonte, PA, USA). Accordingly, a 20 mL glass vial was filled with 3 g FOSs liquid sample (0.2 g in 5 mL distilled water) and 2 g sodium chloride. It was then hermetically capped with a silicon septum. The sample vial was incubated at 45ºC for 15 min in a thermostatic bath to equilibrate the headspace volatiles, which were then extracted under the same conditions for 20 min. Volatiles on the fiber were desorbed in the GC injector at 250ºC for 3 min prior to being separated in a DB-5MS column (30 m length × 0.25 mm i.d. × 0.25 μm film thickness), with helium as the carrier gas at a flow rate of 0.6 mL/min. The separation conditions were performed according to what has been described in the previous study (Hong et al., 2023). Volatile compounds were identified using NIST Mass Spectrometry.

**Reference:** Hong J, Kim M-J, Oh WY, Lee J: Evaluation of deodorization techniques using cyclodextrins on the headspace volatiles and antioxidant properties of onion. Food Chemistry 2023; 410:135416. https://doi.org/10.1016/j.foodchem.2023.135416.

**Table S2** Retention time, slope and R^2^ value for analysis of sugars and FOSs extracted from red onion.

| Carbohydrates | Retention time (min) | Slope | R^2^ |
| --- | --- | --- | --- |
| Fructose | 6.859 | y = 154.97x | 0.9999 |
| Glucose | 7.976 | y = 134.17x | 0.9999 |
| Sucrose | 9.379 | y = 162.97x | 0.9999 |
| Neokestose | 15.679 | y = 137.83x | 0.9994 |
| Kestose | 17.048 | y = 247.11x | 0.9959 |
| Nystose | 24.018 | y = 252.5x | 0.9979 |
| Fructofuranosyl nystose | 33.466 | y = 219.03x | 0.9946 |

**Fig. S3** Calibration curves obtained by plotting the average of mean peak areas and concentrations of sugars and FOSs.

**Table S3** Selected volatile compounds from crude FOSs and purified FOSs.

| RT | Compound | Formular | MW | Base Peak | Crude FOSs | Purified FOSs  (*C. orthopsilosis* FLA44.2) | Purified FOSs  (*P. melissophilus* FLA 48) |
| --- | --- | --- | --- | --- | --- | --- | --- |
| 3.58 | Dimethyl disulfide | C_2_H_6_S_2_ | 93.99 | 94, 79, 45 | 2.48 × 10^7^ | ND | ND |
| 8.39 | Methyl propyl disulfide | C_4_H_10_S_2_ | 122.02 | 122, 80, 43 | 2.79 × 10^7^ | 2.58 × 10^7^ | 3.08 × 10^7^ |
| 8.75 | Methyl 1-propenyl disulfide | C_4_H_8_S_2_ | 120.01 | 120, 73, 45 | 1.34 × 10^7^ | 1.19 × 10^7^ | 1.11 × 10^7^ |
| 9.67 | Dimethyl trisulfide | C_2_H_6_S_3_ | 125.96 | 126, 79, 45 | 4.60 × 10^6^ | 2.14 × 10^6^ | ND |
| 14.54 | Dimethyl tetrasulfide | C_16_H_26_O | 157.94 | 158, 94, 79 | 2.35× 10^6^ | 3.21 × 10^6^ | 2.70 × 10^6^ |
| 14.74 | Dipropyl disulfide | C_2_H_6_S_4_ | 150.05 | 150, 108, 43 | 2.95× 10^6^ | 3.34 × 10^6^ | 3.39 × 10^6^ |
| 19.97 | 1,3-bis(1,1-Dimethylethyl)benzene | C_14_H_22_ | 190.32 | 175, 57, 41 | 4.27× 10^6^ | 2.51 × 10^6^ | 3.28 × 10^6^ |
| 24.12 | Dipropyl trisulfide | C_6_H_14_S_3_ | 182.03 | 182, 75, 43 | 5.10× 10^6^ | 1.06 × 10^6^ | ND |
